# On the origin of cerebral small vessel disease: MRI markers of cSVD in young adults with hypertension

**DOI:** 10.1016/j.cccb.2025.100397

**Published:** 2025-09-20

**Authors:** M.H. Snijders, E. Janssen, E. Verburgt, A. ter Telgte, T.N.A. van den Berg, M.C. Maas, F.J.A. Meijer, A.M. Tuladhar, N.P. Riksen, J. Deinum, F.E. de Leeuw

**Affiliations:** a Department of Neurology, Research Institute for Medical research and Innovation and Donders Institute for Brain, Cognition and Behaviour, Radboud University Nijmegen Medical Centre, PO Box 9101 6500, HB, Nijmegen, Netherlands; b VASCage – Centre on Clinical Stroke Research, Innsbruck, Austria; c Department of Neurology, Medical University of Innsbruck, Innsbruck, Austria; d Department of Internal Medicine, Rijnstate Hospital, Arnhem, Netherlands; e Department of Medical Imaging, Radboud University Medical Center, Nijmegen, Netherlands; f Department of Internal Medicine, Radboud University Medical Center, Nijmegen, Netherlands

**Keywords:** Cerebral small vessel disease, Hypertension, Young adults, MRI, Cerebrovascular disease

## Abstract

**Introduction:**

Cerebral small vessel disease (cSVD) contributes to stroke and cognitive decline, with MRI markers of cSVD increasing with age. Hypertension is an important risk factor for cSVD in older adults but its impact in younger individuals remains less clear. This study investigates whether MRI markers of cSVD are more prevalent in young hypertensive individuals compared to normotensive controls.

**Methods:**

In this cross-sectional study, 60 patients with hypertension and 21 controls aged 18–55 years underwent 3T MRI to assess cSVD markers: white matter hyperintensities (WMH), lacunes, and microbleeds. Group differences were assessed using *t*-tests, chi-square tests, or non-parametric methods. We examined associations between blood pressure and cSVD markers using multivariable regression models, including linear, logistic, ordinal logistic, and penalized logistic regression, adjusting for potential confounders.

**Results:**

Patients with hypertension were older (median (IQR) 35.6 (29.6–41.4) years vs 29.2 (27.8–33.2) years), had a higher BMI, and lower education levels while proportion of females was similar. Deep WMH burden was significantly higher in hypertensive participants (median Fazekas score: 1 [IQR: 0–1] vs 0 [IQR: 0–0]; *p* < 0.001). Hypertension increased odds of deep WMH (OR 5.49, *p* = 0.011). Lacunes and microbleeds were rare and observed only in hypertensive participants. Duration since hypertension diagnosis was not significantly associated with WMH volume (β=7.27, *p* = 0.111) after adjusting for age.

**Conclusion:**

WMH are more prevalent in young adults with hypertension, suggesting early microvascular brain changes. These findings underscore the importance of early detection and treatment of hypertension to potentially prevent long-term cerebrovascular changes.

## Highlights

• cSVD MRI markers are already visible in young patients with hypertension.
• White matter hyperintensities were more prevalent than lacunes and microbleeds.
• High blood pressure should be monitored and treated in adults to prevent damage.
• Studying young patients with hypertension may offer insights into cSVD onset.

## Introduction

Cerebral small vessel disease (cSVD) is a condition affecting small brain blood vessels, resulting in white matter hyperintensities (WMH), microbleeds, and lacunes (so called MRI markers of cSVD) [1]. cSVD is an important cause of both ischemic and haemorrhagic stroke, cognitive decline, and vascular dementia [2]. MRI markers of cSVD are frequently seen in older adults (> 60 years) and the prevalence increases with age [2]. Around 5 % of individuals in their 50s show signs of cSVD and by the age of 90, almost everyone is affected [2]. However, the exact aetiology of the disease remains unclear, as most studies focus on examining these end-stage MRI markers of cSVD in patients over 60 years [3], thereby lacking the opportunity to investigate cSVD onset.

Hypertension, which affected 1.28 billion adults globally in 2023, is the primary modifiable risk factor for cSVD [4]. Although hypertension prevalence increases with age, it also affects younger populations, with approximately 1 in 8 adults under 40 years diagnosed with hypertension [5,6]. While the association between hypertension and cSVD is well-established in older adults, this has rarely been investigated in young individuals with hypertension, providing limited insight into the early stages of cSVD development [[7], [8], [9]]. Studying younger individuals at risk for cSVD, such as individuals with hypertension, provides the opportunity to study cSVD in a much earlier stage, before irreversible brain damage and neurological symptoms are present. In the general population, the frequency of MRI markers of cSVD is low in young individuals [10]. However, several studies have shown that changes in small vessel characteristics, such as density and caliber, and WMH could be observed in adults with cardiovascular risk factors [[11], [12], [13]]. As hypertension is the number one risk factor of cSVD, we however already expect to see first visible signs of MRI markers in these at-risk individuals. We aimed to examine the prevalence and location of MRI markers of cSVD in young individuals with hypertension (aged 18–55 years) compared to normotensive controls.

## Methods

### Study population and design

This study is a cross-sectional study conducted at the Radboud University Medical Center in the Netherlands [14]. Patients with hypertension were recruited from the outpatient clinic of the Department of Internal Medicine at the Radboud University Medical Center in Nijmegen and the Rijnstate Hospital in Arnhem, both in the Netherlands. Recruitment of the normotensive controls involved the social circles of the researchers and the participants with hypertension. The study protocol was approved by the METC region Arnhem-Nijmegen and all participants provided written informed consent.

### Inclusion and exclusion criteria

Patients aged 18–55 years who were referred to the department of Internal Medicine to analyse the cause of their hypertension were eligible for participation. Hypertension was defined as a systolic blood pressure of >140 mmHg/diastolic blood pressure of >90 mmHg within three months before study inclusion, regardless of antihypertensive medication use. Normotensive controls were defined as individuals aged 18–40 years with a blood pressure <120/85 mmHg without any history of antihypertensive treatment.

Participants were excluded if they had contraindications for MRI, such as pregnancy or metal implants. Individuals with a history of a clinically symptomatic ischemic or haemorrhagic stroke, or transient ischemic attack (TIA) were also excluded. In addition, participants with conditions that could result in MRI abnormalities mimicking cSVD were excluded. These included large artery disease (defined as >50 % stenosis of the internal carotid or vertebral arteries, based on ultrasound collected at baseline), cardio-embolism, and vasculitis. Other exclusion criteria were the presence of major neurological or psychiatric disorders or any condition that would prevent long-term follow-up, and the inability to provide written informed consent. Information on possible exclusion criteria was obtained through self-report and medical documentation available in the hospital.

### Study procedures

During the study visit, blood pressure was measured three times while participants were seated, with their feet flat on the floor, after five minutes of rest. Measurements were done in a quiet room with a researcher present using an automatic blood pressure monitor (Dinamap; GE Healthcare, Chicago, IL). In addition, standardized questionnaires were administered to collect information about demographic characteristics, cardiovascular risk factors, and relevant medical history. Educational level was assessed using the revised Verhage classification system [15], which is widely used in Dutch neuropsychological research.

### MRI acquisition and analysis

All participants underwent brain MRI during the study visit using a 3T MRI system (MAGNETOM PrismaFit, Siemens Healthcare, Erlangen, Germany) with a 20-channel head-neck coil. The standardized protocol included high-resolution structural sequences (T1-weighted Magnetization Prepared 2 Rapid Acquisition Gradient Echo (MP2RAGE), Fluid-Attenuated Inversion Recovery (FLAIR), and Susceptibility-Weighted Imaging (SWI). Acquisition parameters are described in the protocol of the Hyperintense Study [14].

#### WMH segmentation and probability map

We employed a deep learning–based segmentation tool, the MIAC Automated Region Segmentation for White Matter Hyperintensities (MARS-WMH) algorithm (available at https://github.com/miac-research/MARS-WMH) to automatically segment WMH using T1-weighted and FLAIR MRI scans [16,17]. All resulting WMH masks were visually inspected for segmentation accuracy and manually corrected when necessary. WMH volume was calculated separately for periventricular WMH (defined as ≤ 10 mm of lateral ventricles) and deep WMH (defined as >10 mm distant to ventricles).

To create the WMH probability map, MRI scans of the patients with hypertension and controls were used separately. The binary WMH mask of each patient was registered to the 1 mm resolution Montreal Neurological Institute (MNI)-152 brain template [18]. To this end, the brain extracted FLAIR image was first coarsely aligned to brain extracted MNI-152 T2-template using linear registration with FSL’s flirt tool [19]. Then, the registration was finetuned using the deformable registration tool SynthMorph [20]. All voxels that were not located in the white matter (using the MNI probabilistic white matter atlas, thresholded at 30 %) were removed and the final result was visually inspected.

To create the probability map in MNI space, all coregistered WMH masks were summed and divided by the total number of patients with hypertension or controls, resulting in a map indicating the probability that each voxel contains WMH or not.

#### Ratings of cSVD markers

MRI markers of cSVD, including WMH, lacunes, and cerebral microbleeds, were visually assessed according to the STRIVE criteria [1]. WMH burden was scored separately for periventricular and deep regions using the Fazekas scale [21]. Lacunes were defines as round or ovoid lesions (3–15 mm in diameter) with CSF-like signal characteristics on T1. Although the STRIVE criteria also include perivascular spaces as a marker of cSVD, these were not assessed in this study since these are best visualized on a T2-weighted MRI sequence, which was not available in this study. Cerebral microbleeds were classified according to their location as follows: lobar (involving the cortex and/or deep white matter regions); basal ganglia/thalamus; and infratentorial (involving the brainstem and/or cerebellum).

The MRI data were independently assessed by two raters (E.J and M.S) to ensure reliability and reduce bias. After the assessments, they discussed the discrepancies and resolved any differences. The Intraclass Correlation Coefficient (ICC) was calculated to assess the level of agreement between two raters for the number of lacunes and microbleeds and the weighted kappa was calculated for the ordinal Fazekas scores. The ICC for lacunes was 1.00, indicating complete agreement between raters. For microbleeds, the ICC was 0.653 (95 % CI: 0.509 – 0.762), indicating good interrater reliability. The weighted kappa for deep WMH was 0.796 (*p* < 0.001), indicating good to excellent reliability. For periventricular WMH, the weighted kappa was 0.447 (*p* < 0.001), which corresponds to moderate reliability.

### Statistical analysis

We assessed the normality of continuous variables within each group using the Shapiro-Wilk test. To evaluate group differences between hypertensive and normotensive individuals, we used independent samples *t*-tests or Mann-Whitney U tests for continuous or ordinal variables, and Chi-square or Fisher’s exact tests for categorical variables, depending on data distribution.

We conducted all regression analyses as multivariable models, adjusting for potential confounders including age, sex, BMI, and education level when data permitted. If variables contained only zero values in one or both groups, or lacked sufficient variability, we did not perform group comparisons or regression analyses and instead reported descriptive statistics.

We applied regression models to examine the association between systolic blood pressure and cSVD markers. For continuous outcomes such as WMH volume, we used linear regression models.

To analyse count-based outcomes, including the number of lacunes and cerebral microbleeds, we initially used negative binomial regression models to account for overdispersion. However, the extremely low prevalence of these markers did not allow meaningful regression analyses, so we reported only descriptive statistics.

For markers measured on ordinal scales, such as Fazekas scores for WMH burden, we first applied ordinal logistic regression. Due to limited variability, particularly in the control group, we could not reliably estimate these models. Consequently, we dichotomized WMH scores and used binary logistic regression instead. Because all controls had a Fazekas score of 0 for periventricular WMH, we employed Firth’s penalized logistic regression.

We examined the association between the duration since hypertension diagnosis (in years) and total WMH volume using linear regression, adjusting for age. We excluded one participant from this analysis due to a rare genetic form of hypertension and an unusually high WMH volume at a young age, which we considered an outlier.

We did not apply multiple comparisons correction, as the conducted analyses were pre-specified and limited to well-established SVD markers. Given the exploratory nature of the study and the small sample size, applying such correction would substantially reduce statistical power. However, not correcting for multiple comparisons may increase the risk of type I error and results should therefore be interpreted with caution.

We conducted all statistical analyses using R software (version 4.4.3) and considered p-values <0.05 statistically significant.

## Results

### Study population

The study included 60 patients with hypertension and 21 normotensive controls. Baseline characteristics are shown in Table 1. Patients with hypertension were older (median (IQR) 35.6 (29.6-41.4) years than the normotensive controls (29.2 (27.8-33.2) years. The proportion of females was similar between groups: 55 % (*n* = 33) in the hypertensive group and 52 % (*n* = 11) in the control group. Both mean systolic and diastolic blood pressure were higher in the hypertensive group (147 mmHg (SD 17.0) and 91 mmHg (SD 11.7)) compared to controls (120 mmHg (SD 10.9) and 75 mmHg (SD 8.3)).

**Table 1:** Baseline characteristics of patients with hypertension and controls.

	Hypertension (*n* = 60)	Control (*n* = 21)	p-value
Age (years, median (IQR))	35.6 (29.6 – 41.4)	29.2 (27.8 – 33.2)	<0.001
Female, n ( %)	33 (55)	11 (52)	1.000
Systolic blood pressure (mmHg, mean (SD))	147 (17.0)	120 (10.9)	<0.001
Diastolic blood pressure (mmHg, mean (SD))	91 (11.7)	75 (8.3)	<0.001
BMI (mean (SD))	28.5 (5.9)	22.7 (2.8)	<0.001
Total Defined Daily Dosage (DDD) (median (IQR)) Antihypertensive medications, n ( %) * Calcium-antagonist RAS-inhibitors ⁎⁎ Diuretic Betablocker Alphablocker	1.5 (1.0 - 2.8) 52 (86.7) 39 (65) 28 (46.6) 17 (28.3) 7 (11.6) 6 [10]	0 (0 – 0) 0 (0) 0 (0) 0 (0) 0 (0) 0 (0) 0 (0)	<0.001 <0.001
Cholesterol lowering medication, n ( %)	6 [10]	0 (0)	0.331
Alcohol use ever, n ( %)	50 (83.3)	21 (100)	0.103
Daily smoking, n ( %)	5 (8.3)	2 (9.5)	1.000
Education level (Verhage classification) (median (IQR))	6.0 (5.0 - 6.3)	7.0 (7.0 - 7.0)	<0.001

⁎ Patients may be counted in more than one category due to use of multiple antihypertensive medications.

⁎⁎ Angiotensin Converting Enzyme (ACE)-inhibitor/Angiotensin Receptor Blocker (ARB).

### MRI markers of cSVD

#### Deep white matter hyperintensities (WMH)

Patients with hypertension had significantly higher Fazekas scores compared to controls (*p* = <0.001) (Table 2). The median (IQR) deep WMH volume in patients with hypertension was 10.9 mm^3^ (1.0 - 43.5), compared to 1.07 mm^3^ (0.0 - 12.4) in controls (*p* = 0.021) (Table 2). Using Firth’s penalised logistic regression, patients with hypertension had significantly higher odds of having deep WMH compared to normotensive controls after adjusting for age, sex, BMI and education level (OR = 5.49, 95 % CI: 1.46 −0 25.5, *p* = 0.011).

**Table 2:** Prevalence of cSVD markers in patients with hypertension and controls. WMH were assessed using the Fazekas score separately for deep and periventricular WMH and as a volumetric measure. Lacunes and microbleeds are shown as number of individuals with lacunes or microbleeds present. Differences between groups were assessed binary logistic regression for deep Fazekas score and Mann-Whitney U tests for WMH volumes.

	Hypertension (*n* = 60)	Control (*n* = 21)	p-value
Deep WMH Fazekas score (0/1/2/3), n ( %) Score 0 Score 1 Score 2 Score 3	25 (41.7) 34 (56.7) 1 (1.7) 0 (0)	18 (85.7) 3 (14.3) 0 (0) 0 (0)	<0.001
Periventricular WMH Fazekas score (0/1/2/3), n ( %) Score 0 Score 1 Score 2 Score 3	49 (81.7) 9 (15.0) 2 (3.3) 0 (0)	21 (100) 0 (0) 0 (0) 0 (0)	NA
WMH volume (mm^3^, median (IQR)) Deep WMH Periventricular WMH Total WMH	10.9 (1.0 - 43.5) 12.0 (1.4 - 66.6) 44.3 (9.4 – 120.0)	1.07 (0.0 – 12.4) 3.2 (0.0 - 24.9) 12.4 (1.93 – 35.8)	0.021 0.164 0.018
Microbleeds present, n ( %)	3 [5]	0 (0)	NA
Lacunes present, n ( %)	1 (1.7)	0 (0)	NA

#### Periventricular white matter hyperintensities (WMH)

Only patients with hypertension showed periventricular WMH with Fazekas scores of 1 or higher (Table 2), limiting statistical comparisons. The median (IQR) periventricular WMH volume in patients with hypertension was 12.0 mm^3^ (1.4 - 66.6), compared to 3.2 mm^3^ (0.0 - 24.9) in controls (*p* = 0.164) (Table 2). Firth’s logistic regression showed a higher odds ratio for the hypertensive group (OR = 3.31, 95 % CI: 0.24–474), but this was not statistically significant (*p* = 0.40).

#### Spatial distribution of WMH

**Fig. 1:**
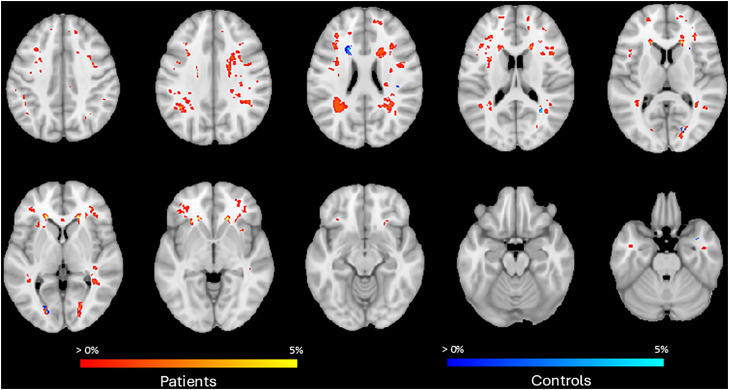
WMH probability map for patients with hypertension and controls. This figure shows the spatial probability of WMH displayed on the MNI-152 template at the voxel level. Red/yellow colours indicate the percentage of patients with hypertension who have a WMH in that voxel, blue the percentage of normotensive controls.

Fig. 1 shows the WMH probability map for the patients with hypertension. Only a small proportion of the white matter was affected by WMH and the probability was low in most regions. The most commonly affected area is the periventricular area, both frontal and parietal. Furthermore, WMH were observed in the centrum semiovale as small punctate lesions.

#### Microbleeds

**Fig. 2:**
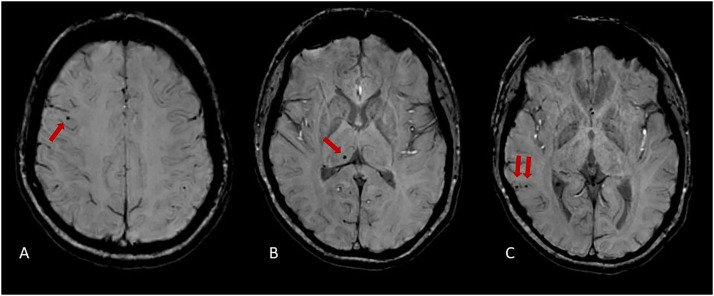
Examples of microbleeds in one patient with hypertension in lobar regions (A, C) and the basal ganglia (B).

A total of 10 microbleeds were observed in 3 patients: one patient had a single infratentorial microbleed; a second patient had 3 microbleeds, all were located lobar; and the third patient had 6 microbleeds, of which 5 were lobar and 1 was in the basal ganglia/thalamus (see Fig. 2). No microbleeds were observed in the control group (Table 2).

#### Lacunes

**Fig. 3:**
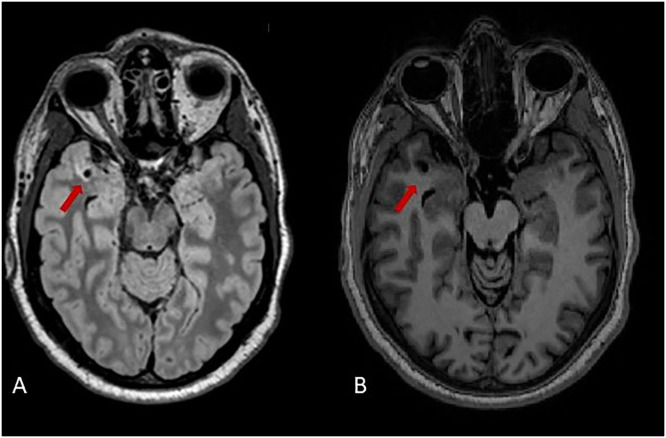
Lacune visible in the temporal lobe in one patient with hypertension, shown on FLAIR (A) and T1 (B).

Only one patient in the hypertensive group had a lacune, located in the temporal lobe, as shown in Fig. 3. No lacunes were observed in the control group (Table 2).

#### Blood pressure and WMH volume

There was no association between systolic blood pressure and WMH volume (β = 5.84, *p* = 0.164). In the same model, age at inclusion (β =−13.08, *p* = 0.139, sex (β =158.64, *p* = 0.139), and BMI (β = 11.26, *p* = 0.429) were not significantly associated with total WMH volume.

#### Duration since hypertension diagnosis and WMH

Duration since hypertension diagnosis was positively associated with WMH volume after adjusting for age (β = 7.27, *p* = 0.111), where β represents the estimated increase in WMH volume (in mm^3^) per additional year of hypertension. However, this association was not statistically significant.

## Discussion

This study investigated the prevalence of cSVD markers in young patients with hypertension compared to normotensive controls. Patients with hypertension had more WMH, especially in the deep WMH, demonstrating that conventional MRI markers of cSVD are already visible in young at-risk patients, offering an interesting population to study early disease mechanisms of cSVD [22]. No significant associations were found between blood pressure or duration since hypertension diagnosis and WMH volume.

These findings are in line with previous research showing an association between hypertension and WMH in older individuals [[7], [8], [9]]. Other studies, like the Framingham Heart Study, have found that higher systolic blood pressure is related to lower white matter integrity in young adults [23,24]. The low prevalence of lacunes and microbleeds we found is also in line with existing literature, which shows that these markers are uncommon before the age of 50 [2]. This indicates that WMH could be the earliest detectable MRI marker of cSVD. However, larger studies are necessary to validate these results. In our cohort, BMI was higher in the individuals with hypertension, making it difficult to separate the independent effect of these variables. Data among older patients with hypertension demonstrated a strong effect of hypertension duration on WMH volume and white matter microstructure [25]. This highlights the cumulative effect of high blood pressure on cerebral microvasculature over time, but our population is likely too young to observe this. Furthermore, the recorded duration may include periods of well-controlled blood pressure due to medication use which we do not account for in this analysis. The actual risk of microvascular damage likely depends on the overall period with hypertension, but also on the quality of blood pressure management during that time.

We observed significant differences in deep WMH but not in periventricular WMH when comparing the Fazekas scores. This is likely due to the strict threshold we applied for rating deep WMH, where the presence of more than one deep WMH lesion was classified as Fazekas 1 according to the original Fazekas classification [21]. Furthermore, the moderate interrater reliability identified for periventricular WMH likely reflects the mild overall WMH burden within this group, resulting in several cases that were challenging to categorise at the lower end of the Fazekas scale. This highlights the limitations of using a categorical rating scale, as it does not fully capture the true WMH burden. The low sensitivity of categorical ratings such as the Fazekas scale likely explains the incongruency between results obtained with visual scores and those obtained quantitatively. In contrast, volumetric measures provide a more accurate reflection of the overall WMH load. When looking at the probability map, WMH in patients with hypertension were mostly located in periventricular areas in parietal and frontal white matter while the overall burden of WMH was relatively low. In normotensive controls, WMH volume was very low and mostly observed in the periventricular WM. This is in line with other studies, describing the periventricular areas as the most commonly affected in cSVD in older individuals, thus supporting the notion that young individuals with hypertension may be a good model to examine early manifestations of cSVD [26,27]Disruption of the anterior thalamic radiation and forceps minor in this area was most strongly associated with cognitive functioning in SVD patients, emphasising the clinical significance of white matter disruption in specific tracts [28].

The low prevalence of cSVD markers such as lacunes and microbleeds, especially in the control group, limited the use of multivariable regression models. However, the absolute differences observed in WMH between patients and controls strongly suggest a higher SVD burden in patients with hypertension. To improve the reliability of the estimates despite the low prevalence of some MRI markers, we used Firth’s penalised logistic regression for the analysis of deep and periventricular WMH. This method reduces bias in maximum likelihood estimates and provides more stable odds ratios when complete separation occurs.

### Clinical implications

The clinical significance of early lesions in the brain of young individuals was not studied, however, similar lesions are linked to cognitive decline and an increased risk of stroke in older adults [2]. Early detection of WMH could be important for risk stratification and preventing neurological complications in young hypertensive patients. Our results highlight that early detection and treatment through routine monitoring of blood pressure in young individuals is important. Recent evidence suggests that maintaining blood pressure below the traditional threshold of 140/90 mmHg in midlife is important for optimal brain health [29].

### Strengths and limitations

An important strength of this study is the use of a young and well-defined population, with strict inclusion and exclusion criteria and standardised MRI acquisition. The reliability of the MRI assessment was good to excellent for most of the cSVD markers. However, the moderate interrater agreement for periventricular WMH suggests that evaluation of this specific marker may be more subjective and less reproducible. Therefore, WMH volumes were also analysed, as these are more objective and thus minimise observer bias.

A limitation is the cross-sectional design, which makes it difficult to draw conclusions about causality and to assess the progression of cSVD over time. Furthermore, perivascular spaces, another important cSVD marker, were not evaluated because T2-weighted imaging was unavailable. In addition, socioeconomic status and genetic predisposition were not studied in this study, which may influence cSVD burden. It should be noted that patients with hypertension included in this study are a selective group of patients with hypertension, as most young individuals with hypertension do not go to the hospital. Finally, selecting normotensive controls from the social circles of researchers and patients may have introduced selection bias, which limits the generalizability of the results.

### Future directions

Future studies should include more advanced imaging techniques such as diffusion tensor imaging (DTI) to assess white matter microstructural integrity or dynamic contrast-enhanced (DCE) MRI to assess blood-brain barrier leakage to provide more information about early pathological processes. Longitudinal studies are needed to determine if early WMH in young individuals with hypertension contribute to more cerebrovascular damage and cognitive decline later in life. Furthermore, further investigating modifiable risk factors, such as diet and physical activity, and how these influence the development of cSVD in young hypertensive individuals could provide important insights into potential targeted preventive strategies for this group.

## Conclusion

In conclusion, this study shows that cSVD is more common in young patients with hypertension than in normotensive controls. This highlights the need for early detection and management of hypertension and other modifiable risk factors to prevent brain damage later in life. Future research should focus on the long-term effects of hypertension on brain health and develop targeted strategies to reduce these effects in younger populations.

## CRediT authorship contribution statement

**M.H. Snijders:** Writing – review & editing, Writing – original draft, Methodology, Formal analysis. **E. Janssen:** Writing – review & editing, Writing – original draft, Supervision, Methodology, Investigation, Formal analysis, Data curation, Conceptualization. **E. Verburgt:** Writing – review & editing, Investigation. **A. ter Telgte:** Writing – review & editing, Conceptualization. **T.N.A. van den Berg:** Writing – review & editing, Investigation. **M.C. Maas:** Writing – review & editing, Methodology. **F.J.A. Meijer:** Methodology. **A.M. Tuladhar:** Writing – review & editing, Supervision. **N.P. Riksen:** Writing – review & editing, Supervision. **J. Deinum:** Writing – review & editing, Supervision. **F.E. de Leeuw:** Writing – review & editing, Writing – original draft, Validation, Supervision, Resources, Methodology, Investigation, Conceptualization.

## Declaration of competing interest

I have nothing to declare.
